# Penta­aqua­bis[4-(2-hydroxy­benzyl­idene­amino)benzene­sulfonato]lead(II)

**DOI:** 10.1107/S1600536809004541

**Published:** 2009-02-13

**Authors:** Xi-Shi Tai, Yi-Min Feng, Zu-Pei Liang

**Affiliations:** a Department of Chemistry and Chemical Engineering, Weifang University, Weifang 261061, People’s Republic of China

## Abstract

In the structure of the title compound, [Pb(C_13_H_10_NO_4_S)_2_(H_2_O)_5_], two S—O bonds and one C—N bond have lengths of 1.421 (9), 1.425 (8) and 1.268 (11) Å, respectively, which suggests they are double bonds. Mol­ecules form a two-dimensional layered structure *via* O—H⋯O and O—H⋯N inter­actions. The Pb atom adopts distorted cubo-octahedral coordination.

## Related literature

For our previous work on the coordination chemistry of aroylhydrazones, see: Tai *et al.* (2003 ▶, 2008 ▶); Tai, Yin & Feng (2007 ▶); Tai, Yin & Kong (2007 ▶); Xi-Shi & Yi-Min (2008 ▶).
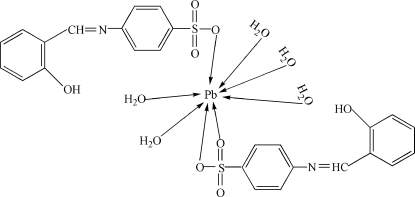


## Experimental

### 

#### Crystal data

[Pb(C_13_H_10_NO_4_S)_2_(H_2_O)_5_]
*M* *_r_* = 849.83
Monoclinic, 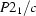

*a* = 35.618 (4) Å
*b* = 7.3407 (10) Å
*c* = 11.6218 (18) Å
β = 99.146 (2)°
*V* = 3000.0 (7) Å^3^
*Z* = 4
Mo *K*α radiation
μ = 5.83 mm^−1^
*T* = 298 K
0.50 × 0.40 × 0.38 mm

#### Data collection

Bruker SMART CCD area-detector diffractometer
Absorption correction: multi-scan (*SADABS*; Bruker, 2000 ▶) *T*_min_ = 0.159, *T*_max_ = 0.215 (expected range = 0.080–0.109)
14411 measured reflections
5264 independent reflections
4635 reflections with *I* > 2σ(*I*)
*R*_int_ = 0.045

#### Refinement

*R*[*F* ^2^ > 2σ(*F* ^2^)] = 0.051
*wR*(*F* ^2^) = 0.129
*S* = 1.09
5264 reflections
397 parameters
H-atom parameters constrained
Δρ_max_ = 1.95 e Å^−3^
Δρ_min_ = −4.10 e Å^−3^

### 

Data collection: *SMART* (Bruker, 2000 ▶); cell refinement: *SAINT* (Bruker, 2000 ▶); data reduction: *SAINT*; program(s) used to solve structure: *SHELXS97* (Sheldrick, 2008 ▶); program(s) used to refine structure: *SHELXL97* (Sheldrick, 2008 ▶); molecular graphics: *SHELXTL* (Sheldrick, 2008 ▶); software used to prepare material for publication: *SHELXTL*.

## Supplementary Material

Crystal structure: contains datablocks global, I. DOI: 10.1107/S1600536809004541/at2722sup1.cif

Structure factors: contains datablocks I. DOI: 10.1107/S1600536809004541/at2722Isup2.hkl

Additional supplementary materials:  crystallographic information; 3D view; checkCIF report

## Figures and Tables

**Table:** 

Pb1—O9	2.523 (7)
Pb1—O5	2.531 (6)
Pb1—O10	2.534 (7)
Pb1—O11	2.576 (7)
Pb1—O12	2.702 (7)
Pb1—O13	2.713 (8)
Pb1—O1	2.761 (8)
Pb1—O2	2.882 (8)

**Table:** 

S1—O1—Pb1	102.3 (4)
S1—O2—Pb1	97.8 (4)

**Table 2:** Hydrogen-bond geometry (Å, °)

*D*—H⋯*A*	*D*—H	H⋯*A*	*D*⋯*A*	*D*—H⋯*A*
O4—H4⋯N1	0.82	1.90	2.626 (9)	147
O4—H4⋯O4^i^	0.82	2.59	2.897 (9)	104
O8—H8⋯N2	0.82	1.88	2.611 (10)	147
O8—H8⋯O8^ii^	0.82	2.60	2.933 (9)	106
O9—H9*A*⋯O6	0.85	2.04	2.781 (11)	146
O9—H9*B*⋯O5^iii^	0.85	2.17	2.911 (9)	146
O10—H10*A*⋯O6^iv^	0.85	2.12	2.914 (9)	156
O10—H10*B*⋯O7^iii^	0.85	1.94	2.771 (9)	167
O11—H11*A*⋯O3^v^	0.85	2.07	2.883 (11)	162
O11—H11*B*⋯O7^iv^	0.85	2.06	2.772 (9)	141
O12—H12*A*⋯O3^v^	0.85	2.03	2.841 (13)	159
O12—H12*B*⋯O2^vi^	0.85	2.08	2.922 (11)	170
O13—H13*A*⋯O2^vi^	0.85	2.54	3.287 (13)	148
O13—H13*B*⋯O1^vii^	0.85	2.23	2.867 (11)	132
C6—H6⋯O1	0.93	2.52	2.898 (10)	104
C15—H15⋯O6	0.93	2.52	2.907 (10)	105

Symmetry codes: (i) 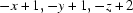; (ii) 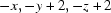; (iii) 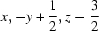; (iv) 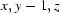; (v) 
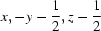
; (vi) 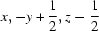; (vii) 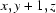.

**Fig. 1.**
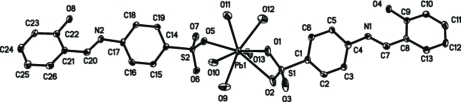
The molecular structure of (I) showing 50% displacement ellipsoids.

## supplementary crystallographic information

### Comment

As part of our ongoing studies of the coordination chemistry of aroylhydrazones ligands (Tai *et al.*, 2003, 2008; Xi-Shi & Yi-Min, 2008; Tai, Yin & Feng, 2007; Tai, Yin & Kong, 2007), we now report the synthesis and structure of the title compound, (I), (Fig. 1).

In the molecule of (I), both C7—N1 [1.268 (11) Å], S1—O2 [1.421 (9) Å] and S1—O3 [1.425 (8) Å] are close to double-bond separations, indicating that the Lewis structure shown in the scheme is only an approximation to the electron distribution in the molecule. Otherwise, the geometrical parameters for (I) are normal (Table 1). The molecules form a two-dimensional layered structure by the O—H···O and O—H···N interactions (Table 2).

### Experimental

The solution of 1.0 mmol 4-(2-hydroxybenzylideneamino)benzene sulfonic acid and 1.0 mmol NaOH in 5 ml 95% ethanol was added to a solution of 0.5 mmol Pb(CH_3_COO)_2_.4H_2_O in 5 ml ethanol at room temperature. The mixture was refluxed for 4 h with stirring, then the resulting precipitate was filtered, washed, and dried *in vacuo* over P_4_O_10_ for 48 h. Single crystals suitable for X-ray structural analysis was obtained by slowly evaporating from methanol at room temperature, which afforded colourless crystals.

### Refinement

The H atoms were placed geometrically [C—H = 0.93 Å, O—H = 0.82 and 0.85 Å] and refined as riding with *U*_iso_(H) = 1.2*U*_eq_(C) or 1.5*U*_eq_(hydroxy and water O).

### Crystal data

**Table:**

[Pb(C_13_H_10_NO_4_S)_2_(H_2_O)_5_]	*F*(000) = 1672
*M**_r_* = 849.83	*D*_x_ = 1.882 Mg m^−^^3^
Monoclinic, *P*2_1_/*c*	Mo *K*α radiation, λ = 0.71073 Å
Hall symbol: -P 2ybc	Cell parameters from 7854 reflections
*a* = 35.618 (4) Å	θ = 2.3–28.2°
*b* = 7.3407 (10) Å	µ = 5.83 mm^−^^1^
*c* = 11.6218 (18) Å	*T* = 298 K
β = 99.146 (2)°	Block, colourless
*V* = 3000.0 (7) Å^3^	0.50 × 0.40 × 0.38 mm
*Z* = 4	

### Data collection

**Table:**

Bruker SMART CCD area-detector diffractometer	5264 independent reflections
Radiation source: fine-focus sealed tube	4635 reflections with *I* > 2σ(*I*)
graphite	*R*_int_ = 0.045
φ and ω scans	θ_max_ = 25.0°, θ_min_ = 2.8°
Absorption correction: multi-scan (*SADABS*; Bruker, 2000)	*h* = −38→42
*T*_min_ = 0.159, *T*_max_ = 0.215	*k* = −8→8
14411 measured reflections	*l* = −12→13

### Refinement

**Table:**

Refinement on *F*^2^	Primary atom site location: structure-invariant direct methods
Least-squares matrix: full	Secondary atom site location: difference Fourier map
*R*[*F*^2^ > 2σ(*F*^2^)] = 0.051	Hydrogen site location: inferred from neighbouring sites
*wR*(*F*^2^) = 0.129	H-atom parameters constrained
*S* = 1.09	*w* = 1/[σ^2^(*F*_o_^2^) + (0.0518*P*)^2^ + 32.8197*P*] where *P* = (*F*_o_^2^ + 2*F*_c_^2^)/3
5264 reflections	(Δ/σ)_max_ = 0.001
397 parameters	Δρ_max_ = 1.95 e Å^−^^3^
0 restraints	Δρ_min_ = −4.10 e Å^−^^3^

### Special details

**Table:**

Geometry. All e.s.d.'s (except the e.s.d. in the dihedral angle between two l.s. planes) are estimated using the full covariance matrix. The cell e.s.d.'s are taken into account individually in the estimation of e.s.d.'s in distances, angles and torsion angles; correlations between e.s.d.'s in cell parameters are only used when they are defined by crystal symmetry. An approximate (isotropic) treatment of cell e.s.d.'s is used for estimating e.s.d.'s involving l.s. planes.
Refinement. Refinement of *F*^2^ against ALL reflections. The weighted *R*-factor *wR* and goodness of fit *S* are based on *F*^2^, conventional *R*-factors *R* are based on *F*, with *F* set to zero for negative *F*^2^. The threshold expression of *F*^2^ > σ(*F*^2^) is used only for calculating *R*-factors(gt) *etc*. and is not relevant to the choice of reflections for refinement. *R*-factors based on *F*^2^ are statistically about twice as large as those based on *F*, and *R*- factors based on ALL data will be even larger.

### Fractional atomic coordinates and isotropic or equivalent isotropic displacement parameters (Å^2^)

**Table:**

	*x*	*y*	*z*	*U*_iso_*/*U*_eq_	
Pb1	0.254090 (10)	0.60871 (5)	0.70033 (3)	0.02876 (13)	
N1	0.4786 (2)	0.3808 (10)	0.7656 (6)	0.0272 (17)	
N2	0.0183 (2)	0.8703 (10)	0.7901 (6)	0.0300 (18)	
O1	0.29367 (18)	0.3019 (11)	0.6522 (7)	0.0485 (19)	
O2	0.3039 (2)	0.5492 (12)	0.5314 (8)	0.059 (2)	
O3	0.3111 (2)	0.2452 (14)	0.4657 (8)	0.071 (3)	
O4	0.53202 (17)	0.4584 (10)	0.9417 (5)	0.0378 (16)	
H4	0.5103	0.4481	0.9062	0.057*	
O5	0.20067 (17)	0.7699 (8)	0.7789 (5)	0.0307 (14)	
O6	0.18530 (18)	1.0295 (9)	0.6507 (5)	0.0316 (14)	
O7	0.19465 (18)	1.0692 (9)	0.8588 (6)	0.0359 (15)	
O8	−0.03384 (18)	0.9558 (10)	0.9153 (5)	0.0377 (16)	
H8	−0.0123	0.9362	0.9013	0.057*	
O9	0.2255 (3)	0.7960 (11)	0.5263 (6)	0.063 (2)	
H9A	0.2062	0.8562	0.5397	0.076*	
H9B	0.2194	0.7307	0.4658	0.076*	
O10	0.2010 (2)	0.4078 (9)	0.5991 (5)	0.0408 (17)	
H10A	0.2025	0.3012	0.6280	0.049*	
H10B	0.2016	0.4010	0.5264	0.049*	
O11	0.2377 (2)	0.3831 (9)	0.8548 (6)	0.0445 (18)	
H11A	0.2571	0.3215	0.8837	0.053*	
H11B	0.2202	0.3106	0.8253	0.053*	
O12	0.3084 (2)	0.6237 (10)	0.8910 (6)	0.052 (2)	
H12A	0.3089	0.5257	0.9302	0.062*	
H12B	0.3046	0.7129	0.9344	0.062*	
O13	0.2833 (2)	0.9472 (11)	0.7455 (8)	0.058 (2)	
H13A	0.2818	0.9843	0.8139	0.069*	
H13B	0.2740	1.0255	0.6951	0.069*	
S1	0.31440 (6)	0.3667 (3)	0.56240 (19)	0.0272 (5)	
S2	0.18201 (6)	0.9476 (3)	0.76156 (17)	0.0231 (4)	
C1	0.3629 (2)	0.3685 (11)	0.6230 (7)	0.0237 (18)	
C2	0.3894 (3)	0.4201 (13)	0.5545 (7)	0.028 (2)	
H2	0.3812	0.4521	0.4771	0.034*	
C3	0.4275 (2)	0.4250 (13)	0.5985 (7)	0.030 (2)	
H3	0.4451	0.4616	0.5518	0.036*	
C4	0.4396 (2)	0.3745 (12)	0.7138 (8)	0.0269 (19)	
C5	0.4133 (2)	0.3274 (13)	0.7839 (7)	0.0269 (19)	
H5	0.4215	0.2999	0.8620	0.032*	
C6	0.3745 (2)	0.3204 (13)	0.7387 (7)	0.030 (2)	
H6	0.3569	0.2843	0.7852	0.036*	
C7	0.5050 (3)	0.3422 (13)	0.7080 (8)	0.029 (2)	
H7	0.4983	0.3024	0.6315	0.035*	
C8	0.5449 (2)	0.3570 (12)	0.7553 (7)	0.0233 (18)	
C9	0.5573 (2)	0.4157 (12)	0.8698 (7)	0.0258 (19)	
C10	0.5957 (2)	0.4223 (13)	0.9124 (7)	0.029 (2)	
H10	0.6039	0.4594	0.9888	0.035*	
C11	0.6218 (3)	0.3746 (13)	0.8431 (9)	0.035 (2)	
H11	0.6476	0.3820	0.8725	0.042*	
C12	0.6104 (3)	0.3158 (14)	0.7307 (8)	0.034 (2)	
H12	0.6284	0.2837	0.6845	0.041*	
C13	0.5724 (3)	0.3050 (12)	0.6873 (8)	0.029 (2)	
H13	0.5647	0.2626	0.6118	0.035*	
C14	0.1336 (2)	0.9073 (11)	0.7639 (7)	0.0232 (18)	
C15	0.1065 (2)	0.9479 (12)	0.6678 (7)	0.0259 (19)	
H15	0.1143	0.9861	0.5989	0.031*	
C16	0.0686 (3)	0.9325 (13)	0.6734 (8)	0.029 (2)	
H16	0.0507	0.9622	0.6088	0.035*	
C17	0.0565 (2)	0.8722 (11)	0.7757 (7)	0.0233 (18)	
C18	0.0838 (2)	0.8274 (13)	0.8711 (7)	0.0268 (19)	
H18	0.0761	0.7855	0.9392	0.032*	
C19	0.1220 (2)	0.8443 (13)	0.8661 (7)	0.0263 (19)	
H19	0.1400	0.8139	0.9303	0.032*	
C20	−0.0091 (3)	0.8373 (12)	0.7066 (8)	0.0279 (19)	
H20	−0.0029	0.7985	0.6358	0.034*	
C21	−0.0482 (2)	0.8562 (12)	0.7154 (7)	0.0249 (19)	
C22	−0.0593 (3)	0.9194 (12)	0.8203 (7)	0.0264 (19)	
C23	−0.0983 (3)	0.9433 (13)	0.8238 (8)	0.031 (2)	
H23	−0.1062	0.9860	0.8915	0.037*	
C24	−0.1247 (3)	0.9040 (13)	0.7285 (9)	0.035 (2)	
H24	−0.1503	0.9207	0.7327	0.042*	
C25	−0.1145 (3)	0.8408 (14)	0.6267 (9)	0.037 (2)	
H25	−0.1330	0.8145	0.5628	0.045*	
C26	−0.0766 (3)	0.8169 (13)	0.6208 (8)	0.032 (2)	
H26	−0.0695	0.7736	0.5521	0.039*	

### Atomic displacement parameters (Å^2^)

**Table:**

	*U*^11^	*U*^22^	*U*^33^	*U*^12^	*U*^13^	*U*^23^
Pb1	0.0247 (2)	0.0297 (2)	0.03319 (19)	0.00055 (15)	0.00855 (13)	−0.00043 (15)
N1	0.024 (4)	0.032 (5)	0.024 (3)	0.000 (3)	0.000 (3)	0.001 (3)
N2	0.020 (4)	0.033 (5)	0.038 (4)	0.001 (3)	0.008 (3)	0.002 (3)
O1	0.020 (3)	0.059 (5)	0.068 (5)	0.004 (3)	0.010 (3)	0.007 (4)
O2	0.023 (4)	0.064 (6)	0.087 (6)	0.011 (4)	0.003 (4)	0.022 (5)
O3	0.022 (4)	0.099 (7)	0.086 (6)	0.004 (4)	−0.006 (4)	−0.060 (6)
O4	0.018 (3)	0.064 (5)	0.031 (3)	0.004 (3)	0.003 (3)	−0.007 (3)
O5	0.027 (3)	0.026 (3)	0.042 (3)	0.006 (3)	0.014 (3)	0.008 (3)
O6	0.033 (4)	0.029 (4)	0.035 (3)	0.000 (3)	0.014 (3)	0.010 (3)
O7	0.030 (4)	0.036 (4)	0.043 (4)	−0.004 (3)	0.010 (3)	−0.004 (3)
O8	0.024 (3)	0.060 (5)	0.030 (3)	−0.005 (3)	0.008 (3)	−0.008 (3)
O9	0.106 (7)	0.050 (5)	0.033 (4)	0.023 (5)	0.010 (4)	0.002 (4)
O10	0.053 (5)	0.037 (4)	0.029 (3)	−0.008 (3)	−0.004 (3)	0.005 (3)
O11	0.043 (4)	0.038 (4)	0.049 (4)	−0.008 (3)	−0.002 (3)	0.012 (3)
O12	0.061 (5)	0.046 (5)	0.044 (4)	−0.009 (4)	−0.005 (4)	−0.003 (3)
O13	0.054 (5)	0.040 (5)	0.076 (5)	−0.012 (4)	−0.001 (4)	−0.003 (4)
S1	0.0165 (10)	0.0292 (13)	0.0351 (11)	0.0024 (9)	0.0015 (9)	−0.0059 (9)
S2	0.0201 (10)	0.0231 (11)	0.0276 (10)	0.0001 (9)	0.0078 (8)	0.0026 (9)
C1	0.021 (4)	0.020 (5)	0.030 (4)	0.001 (4)	0.005 (3)	−0.007 (4)
C2	0.028 (5)	0.034 (5)	0.021 (4)	0.003 (4)	0.000 (4)	0.003 (4)
C3	0.019 (4)	0.040 (6)	0.031 (5)	−0.002 (4)	0.005 (4)	0.006 (4)
C4	0.021 (4)	0.024 (5)	0.034 (5)	−0.004 (4)	0.001 (4)	−0.003 (4)
C5	0.020 (4)	0.037 (5)	0.024 (4)	0.006 (4)	0.002 (3)	−0.001 (4)
C6	0.022 (5)	0.036 (5)	0.032 (5)	0.002 (4)	0.008 (4)	0.009 (4)
C7	0.029 (5)	0.028 (5)	0.029 (4)	−0.001 (4)	0.002 (4)	0.001 (4)
C8	0.019 (4)	0.021 (5)	0.029 (4)	0.003 (4)	−0.001 (3)	0.002 (3)
C9	0.021 (4)	0.029 (5)	0.028 (4)	0.000 (4)	0.008 (4)	0.004 (4)
C10	0.024 (5)	0.038 (6)	0.026 (4)	−0.004 (4)	0.003 (4)	0.000 (4)
C11	0.022 (5)	0.032 (6)	0.051 (6)	0.000 (4)	0.002 (4)	0.009 (4)
C12	0.022 (5)	0.040 (6)	0.044 (5)	0.006 (4)	0.013 (4)	0.001 (5)
C13	0.033 (5)	0.024 (5)	0.032 (5)	0.006 (4)	0.008 (4)	−0.002 (4)
C14	0.023 (4)	0.019 (4)	0.030 (4)	0.003 (4)	0.011 (3)	−0.002 (3)
C15	0.026 (5)	0.029 (5)	0.024 (4)	0.004 (4)	0.007 (3)	0.005 (4)
C16	0.025 (5)	0.031 (5)	0.030 (4)	−0.004 (4)	−0.005 (4)	0.002 (4)
C17	0.017 (4)	0.018 (5)	0.037 (5)	0.003 (3)	0.011 (4)	−0.002 (4)
C18	0.025 (5)	0.031 (5)	0.026 (4)	−0.004 (4)	0.008 (4)	0.004 (4)
C19	0.025 (5)	0.031 (5)	0.022 (4)	0.005 (4)	0.003 (3)	0.004 (4)
C20	0.031 (5)	0.024 (5)	0.030 (4)	0.003 (4)	0.010 (4)	0.000 (4)
C21	0.022 (4)	0.021 (5)	0.032 (4)	−0.001 (4)	0.007 (4)	0.004 (4)
C22	0.030 (5)	0.020 (5)	0.029 (4)	−0.006 (4)	0.006 (4)	0.001 (4)
C23	0.025 (5)	0.036 (5)	0.035 (5)	0.000 (4)	0.013 (4)	0.000 (4)
C24	0.027 (5)	0.030 (5)	0.050 (6)	−0.006 (4)	0.012 (4)	0.006 (4)
C25	0.036 (6)	0.033 (6)	0.042 (5)	−0.019 (5)	0.005 (4)	−0.003 (4)
C26	0.040 (6)	0.030 (5)	0.028 (4)	−0.004 (5)	0.008 (4)	−0.001 (4)

### Geometric parameters (Å, °)

**Table:**

Pb1—O9	2.523 (7)	C3—H3	0.9300
Pb1—O5	2.531 (6)	C4—C5	1.378 (12)
Pb1—O10	2.534 (7)	C5—C6	1.400 (12)
Pb1—O11	2.576 (7)	C5—H5	0.9300
Pb1—O12	2.702 (7)	C6—H6	0.9300
Pb1—O13	2.713 (8)	C7—C8	1.443 (12)
Pb1—O1	2.761 (8)	C7—H7	0.9300
Pb1—O2	2.882 (8)	C8—C9	1.402 (12)
N1—C7	1.268 (11)	C8—C13	1.407 (12)
N1—C4	1.426 (11)	C9—C10	1.380 (12)
N2—C20	1.284 (12)	C10—C11	1.369 (13)
N2—C17	1.398 (11)	C10—H10	0.9300
O1—S1	1.452 (7)	C11—C12	1.373 (14)
O2—S1	1.421 (9)	C11—H11	0.9300
O3—S1	1.425 (8)	C12—C13	1.370 (13)
O4—C9	1.358 (10)	C12—H12	0.9300
O4—H4	0.8200	C13—H13	0.9300
O5—S2	1.463 (6)	C14—C15	1.386 (12)
O6—S2	1.443 (6)	C14—C19	1.397 (12)
O7—S2	1.454 (7)	C15—C16	1.369 (12)
O8—C22	1.340 (10)	C15—H15	0.9300
O8—H8	0.8200	C16—C17	1.399 (12)
O9—H9A	0.8501	C16—H16	0.9300
O9—H9B	0.8500	C17—C18	1.392 (12)
O10—H10A	0.8500	C18—C19	1.377 (12)
O10—H10B	0.8500	C18—H18	0.9300
O11—H11A	0.8500	C19—H19	0.9300
O11—H11B	0.8501	C20—C21	1.422 (12)
O12—H12A	0.8500	C20—H20	0.9300
O12—H12B	0.8500	C21—C26	1.399 (12)
O13—H13A	0.8500	C21—C22	1.418 (12)
O13—H13B	0.8500	C22—C23	1.407 (12)
S1—C1	1.759 (9)	C23—C24	1.364 (13)
S2—C14	1.754 (9)	C23—H23	0.9300
C1—C2	1.379 (12)	C24—C25	1.372 (14)
C1—C6	1.388 (12)	C24—H24	0.9300
C2—C3	1.376 (12)	C25—C26	1.376 (14)
C2—H2	0.9300	C25—H25	0.9300
C3—C4	1.391 (12)	C26—H26	0.9300
			
O9—Pb1—O5	78.7 (2)	C4—C3—H3	120.5
O9—Pb1—O10	76.6 (3)	C5—C4—C3	120.2 (8)
O5—Pb1—O10	83.8 (2)	C5—C4—N1	117.8 (8)
O9—Pb1—O11	143.3 (3)	C3—C4—N1	121.9 (8)
O5—Pb1—O11	77.4 (2)	C4—C5—C6	120.6 (8)
O10—Pb1—O11	73.4 (2)	C4—C5—H5	119.7
O9—Pb1—O12	141.3 (3)	C6—C5—H5	119.7
O5—Pb1—O12	99.5 (2)	C1—C6—C5	118.6 (8)
O10—Pb1—O12	142.0 (2)	C1—C6—H6	120.7
O11—Pb1—O12	70.5 (2)	C5—C6—H6	120.7
O9—Pb1—O13	75.5 (3)	N1—C7—C8	123.3 (8)
O5—Pb1—O13	77.9 (2)	N1—C7—H7	118.4
O10—Pb1—O13	149.1 (2)	C8—C7—H7	118.4
O11—Pb1—O13	125.2 (2)	C9—C8—C13	118.2 (8)
O12—Pb1—O13	66.5 (2)	C9—C8—C7	121.8 (8)
O9—Pb1—O1	115.9 (2)	C13—C8—C7	119.9 (8)
O5—Pb1—O1	153.1 (2)	O4—C9—C10	119.1 (8)
O10—Pb1—O1	78.4 (2)	O4—C9—C8	121.0 (8)
O11—Pb1—O1	78.2 (2)	C10—C9—C8	119.8 (8)
O12—Pb1—O1	83.0 (2)	C11—C10—C9	120.4 (8)
O13—Pb1—O1	126.4 (2)	C11—C10—H10	119.8
O9—Pb1—O2	75.6 (2)	C9—C10—H10	119.8
O5—Pb1—O2	153.7 (2)	C10—C11—C12	121.0 (9)
O10—Pb1—O2	95.2 (2)	C10—C11—H11	119.5
O11—Pb1—O2	127.6 (2)	C12—C11—H11	119.5
O12—Pb1—O2	97.3 (2)	C13—C12—C11	119.5 (9)
O13—Pb1—O2	90.7 (3)	C13—C12—H12	120.2
O1—Pb1—O2	49.4 (2)	C11—C12—H12	120.2
C7—N1—C4	121.6 (7)	C12—C13—C8	121.0 (8)
C20—N2—C17	123.3 (8)	C12—C13—H13	119.5
S1—O1—Pb1	102.3 (4)	C8—C13—H13	119.5
S1—O2—Pb1	97.8 (4)	C15—C14—C19	119.6 (8)
C9—O4—H4	109.5	C15—C14—S2	120.7 (6)
S2—O5—Pb1	135.9 (3)	C19—C14—S2	119.5 (7)
C22—O8—H8	109.5	C16—C15—C14	120.7 (8)
Pb1—O9—H9A	111.8	C16—C15—H15	119.7
Pb1—O9—H9B	111.9	C14—C15—H15	119.7
H9A—O9—H9B	109.8	C15—C16—C17	120.3 (8)
Pb1—O10—H10A	111.0	C15—C16—H16	119.8
Pb1—O10—H10B	111.2	C17—C16—H16	119.8
H10A—O10—H10B	109.2	C18—C17—N2	118.2 (7)
Pb1—O11—H11A	110.9	C18—C17—C16	118.8 (8)
Pb1—O11—H11B	110.8	N2—C17—C16	122.7 (8)
H11A—O11—H11B	109.0	C19—C18—C17	121.0 (8)
Pb1—O12—H12A	110.9	C19—C18—H18	119.5
Pb1—O12—H12B	110.9	C17—C18—H18	119.5
H12A—O12—H12B	109.0	C18—C19—C14	119.5 (8)
Pb1—O13—H13A	113.2	C18—C19—H19	120.2
Pb1—O13—H13B	113.0	C14—C19—H19	120.2
H13A—O13—H13B	110.6	N2—C20—C21	124.2 (8)
O2—S1—O3	113.8 (6)	N2—C20—H20	117.9
O2—S1—O1	110.4 (5)	C21—C20—H20	117.9
O3—S1—O1	112.0 (5)	C26—C21—C22	118.6 (8)
O2—S1—C1	107.2 (4)	C26—C21—C20	121.1 (8)
O3—S1—C1	105.9 (4)	C22—C21—C20	120.3 (8)
O1—S1—C1	107.1 (4)	O8—C22—C23	119.6 (8)
O6—S2—O7	112.3 (4)	O8—C22—C21	121.9 (8)
O6—S2—O5	113.3 (4)	C23—C22—C21	118.5 (8)
O7—S2—O5	111.2 (4)	C24—C23—C22	120.4 (8)
O6—S2—C14	107.7 (4)	C24—C23—H23	119.8
O7—S2—C14	106.0 (4)	C22—C23—H23	119.8
O5—S2—C14	105.9 (4)	C23—C24—C25	122.0 (9)
C2—C1—C6	120.2 (8)	C23—C24—H24	119.0
C2—C1—S1	119.2 (7)	C25—C24—H24	119.0
C6—C1—S1	120.5 (7)	C24—C25—C26	118.8 (9)
C3—C2—C1	121.2 (8)	C24—C25—H25	120.6
C3—C2—H2	119.4	C26—C25—H25	120.6
C1—C2—H2	119.4	C25—C26—C21	121.7 (9)
C2—C3—C4	119.1 (8)	C25—C26—H26	119.1
C2—C3—H3	120.5	C21—C26—H26	119.1
			
O9—Pb1—O1—S1	41.2 (5)	C4—C5—C6—C1	−2.4 (14)
O5—Pb1—O1—S1	159.5 (3)	C4—N1—C7—C8	176.4 (8)
O10—Pb1—O1—S1	110.0 (4)	N1—C7—C8—C9	0.1 (14)
O11—Pb1—O1—S1	−174.8 (4)	N1—C7—C8—C13	177.6 (9)
O12—Pb1—O1—S1	−103.3 (4)	C13—C8—C9—O4	−176.4 (8)
O13—Pb1—O1—S1	−49.5 (5)	C7—C8—C9—O4	1.1 (13)
O2—Pb1—O1—S1	2.6 (3)	C13—C8—C9—C10	0.4 (13)
O9—Pb1—O2—S1	−147.3 (5)	C7—C8—C9—C10	178.0 (8)
O5—Pb1—O2—S1	−158.9 (4)	O4—C9—C10—C11	178.0 (9)
O10—Pb1—O2—S1	−72.5 (4)	C8—C9—C10—C11	1.0 (14)
O11—Pb1—O2—S1	0.5 (5)	C9—C10—C11—C12	−1.3 (15)
O12—Pb1—O2—S1	71.5 (4)	C10—C11—C12—C13	0.1 (15)
O13—Pb1—O2—S1	137.9 (4)	C11—C12—C13—C8	1.4 (15)
O1—Pb1—O2—S1	−2.7 (3)	C9—C8—C13—C12	−1.7 (14)
O9—Pb1—O5—S2	−33.1 (5)	C7—C8—C13—C12	−179.3 (9)
O10—Pb1—O5—S2	−110.6 (5)	O6—S2—C14—C15	2.0 (8)
O11—Pb1—O5—S2	175.1 (6)	O7—S2—C14—C15	122.4 (7)
O12—Pb1—O5—S2	107.6 (5)	O5—S2—C14—C15	−119.4 (7)
O13—Pb1—O5—S2	44.4 (5)	O6—S2—C14—C19	−173.9 (7)
O1—Pb1—O5—S2	−159.1 (4)	O7—S2—C14—C19	−53.6 (8)
O2—Pb1—O5—S2	−21.5 (9)	O5—S2—C14—C19	64.7 (8)
Pb1—O2—S1—O3	131.0 (4)	C19—C14—C15—C16	2.1 (13)
Pb1—O2—S1—O1	4.1 (5)	S2—C14—C15—C16	−173.9 (7)
Pb1—O2—S1—C1	−112.2 (3)	C14—C15—C16—C17	−1.1 (14)
Pb1—O1—S1—O2	−4.3 (5)	C20—N2—C17—C18	−151.5 (9)
Pb1—O1—S1—O3	−132.3 (4)	C20—N2—C17—C16	34.4 (13)
Pb1—O1—S1—C1	112.1 (3)	C15—C16—C17—C18	−0.4 (13)
Pb1—O5—S2—O6	24.2 (7)	C15—C16—C17—N2	173.7 (8)
Pb1—O5—S2—O7	−103.3 (5)	N2—C17—C18—C19	−173.4 (8)
Pb1—O5—S2—C14	142.0 (5)	C16—C17—C18—C19	0.9 (14)
O2—S1—C1—C2	−63.3 (8)	C17—C18—C19—C14	0.0 (14)
O3—S1—C1—C2	58.5 (9)	C15—C14—C19—C18	−1.5 (13)
O1—S1—C1—C2	178.2 (7)	S2—C14—C19—C18	174.5 (7)
O2—S1—C1—C6	116.0 (8)	C17—N2—C20—C21	−172.0 (8)
O3—S1—C1—C6	−122.1 (8)	N2—C20—C21—C26	−179.7 (9)
O1—S1—C1—C6	−2.5 (9)	N2—C20—C21—C22	1.6 (14)
C6—C1—C2—C3	0.1 (14)	C26—C21—C22—O8	178.5 (8)
S1—C1—C2—C3	179.4 (7)	C20—C21—C22—O8	−2.9 (13)
C1—C2—C3—C4	0.9 (14)	C26—C21—C22—C23	−1.2 (13)
C2—C3—C4—C5	−2.6 (14)	C20—C21—C22—C23	177.4 (8)
C2—C3—C4—N1	−178.4 (8)	O8—C22—C23—C24	−178.9 (9)
C7—N1—C4—C5	148.1 (9)	C21—C22—C23—C24	0.8 (14)
C7—N1—C4—C3	−36.0 (13)	C22—C23—C24—C25	0.0 (15)
C3—C4—C5—C6	3.4 (14)	C23—C24—C25—C26	−0.2 (15)
N1—C4—C5—C6	179.4 (8)	C24—C25—C26—C21	−0.3 (15)
C2—C1—C6—C5	0.7 (14)	C22—C21—C26—C25	1.0 (14)
S1—C1—C6—C5	−178.7 (7)	C20—C21—C26—C25	−177.6 (9)

### Hydrogen-bond geometry (Å, °)

**Table:**

*D*—H···*A*	*D*—H	H···*A*	*D*···*A*	*D*—H···*A*
O4—H4···N1	0.82	1.90	2.626 (9)	147
O4—H4···O4^i^	0.82	2.59	2.897 (9)	104
O8—H8···N2	0.82	1.88	2.611 (10)	147
O8—H8···O8^ii^	0.82	2.60	2.933 (9)	106
O9—H9A···O6	0.85	2.04	2.781 (11)	146
O9—H9B···O5^iii^	0.85	2.17	2.911 (9)	146
O10—H10A···O6^iv^	0.85	2.12	2.914 (9)	156
O10—H10B···O7^iii^	0.85	1.94	2.771 (9)	167
O11—H11A···O3^v^	0.85	2.07	2.883 (11)	162
O11—H11B···O7^iv^	0.85	2.06	2.772 (9)	141
O12—H12A···O3^v^	0.85	2.03	2.841 (13)	159
O12—H12B···O2^vi^	0.85	2.08	2.922 (11)	170
O13—H13A···O2^vi^	0.85	2.54	3.287 (13)	148
O13—H13B···O1^vii^	0.85	2.23	2.867 (11)	132
C6—H6···O1	0.93	2.52	2.898 (10)	104
C15—H15···O6	0.93	2.52	2.907 (10)	105

Symmetry codes: (i) −*x*+1, −*y*+1, −*z*+2; (ii) −*x*, −*y*+2, −*z*+2; (iii) *x*, −*y*+1/2, *z*−3/2; (iv) *x*, *y*−1, *z*; (v) *x*, −*y*−1/2, *z*−1/2; (vi) *x*, −*y*+1/2, *z*−1/2; (vii) *x*, *y*+1, *z*.
